# Myelopathy Improvement Following Removal of Cervical Sublaminar Wiring

**DOI:** 10.7759/cureus.2191

**Published:** 2018-02-14

**Authors:** Hurtis J Tullos, Robert G Briggs, Andrew K Conner, Allison E Williams, John B Maxwell, Michael D Martin

**Affiliations:** 1 Department of Neurosurgery, University of Oklahoma Health Sciences Center

**Keywords:** cervical spine, cervical myelopathy, sublaminar wire, wire complication

## Abstract

Posterior cervical wiring has been used by spine surgeons in fixation procedures for patients with spinal instability. It is historically considered an effective method of treating cervical instability with a low risk of complications leading to neurological deterioration. We experienced a case of delayed neurological decline associated with myelopathy, lower extremity spasticity, and associated syringomyelia secondary to instrumentation failure and resultant sublaminar wire protrusion into the cervical spinal cord. In the present case, the construct was removed and the patient underwent a durotomy repair and a posterior fusion of cervical levels 1 and 2 via screw placement under image guidance with a subsequent functional improvement back to baseline. We report this case and review the literature on the complications associated with cervical wire fusion and the methods of minimizing these risks.

## Introduction

Posterior cervical wiring was first introduced by Hadra in treatment for patients with Pott’s disease in 1891, and several variants of the technique have since been introduced [1-4]. Although largely supplanted by the use of different forms of rigid fixation, this technique is still a useful tool in the spinal surgeon’s armamentarium. The risks, including cable migration and neurological decline, are relatively low but certainly present [5-6]. We report a case of myelopathy associated with syringomyelia secondary to instrumentation failure and sublaminar wire protrusion into the cervical spinal cord and review literature regarding this complication.

## Case presentation

A 78-year-old man with a history of cervical fracture repaired via sublaminar wiring following a motorcycle collision in 2006 presented to our service with complaints of progressive paresthesias and difficulty picking up items with his left arm. Associated symptoms included intermittent left leg jerks. No triggers, aggravating factors, or alleviating factors were identified. His symptoms began approximately five years after the original operation.

**Figure 1:**
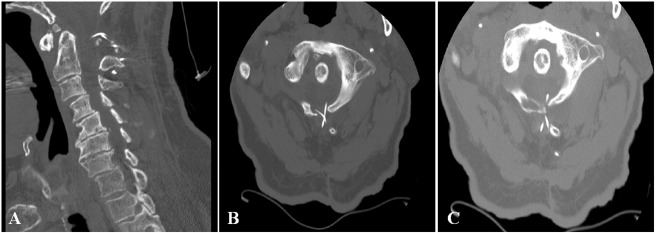
Preoperative Cervical CT Scan Cervical computed tomographic (CT) scan without contrast demonstrating sublaminar wiring in (A) sagittal and (B, C) axial views. On inspection, it appears as if the upper cervical wiring has encroached into the spinal canal with concern for dural and cord penetration.

**Figure 2:**
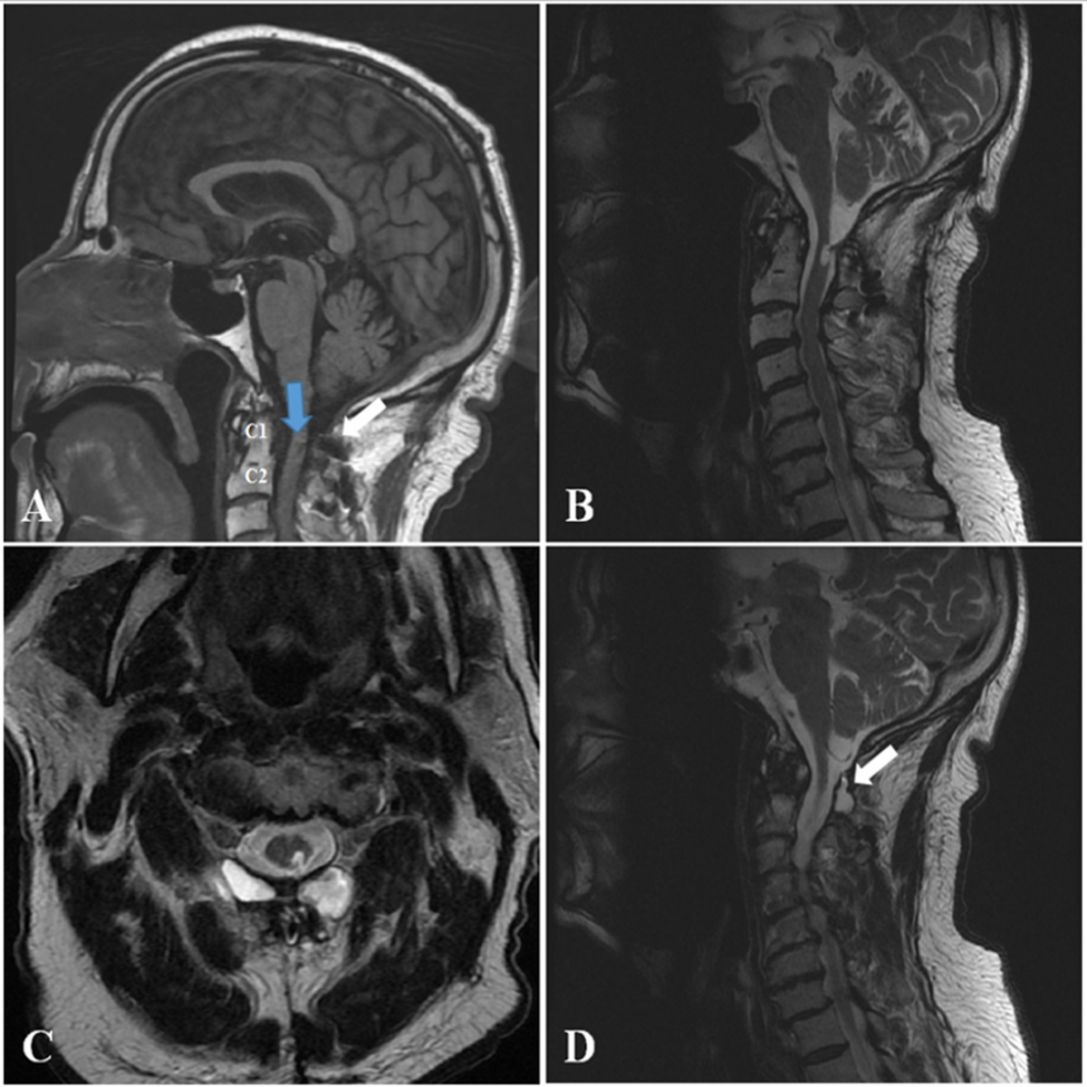
Preoperative Cervical MRI Scan Preoperative magnetic resonance imaging (MRI) scan demonstrating T1 and T2 changes in the cervical spinal canal. (A) T1-weighted non-contrast image of the cervical spinal cord demonstrating a pseudomeningocele (white arrow) and intensity change in the cord between levels C1 and C2 (blue arrow). T2-weighted non-contrast imaging also demonstrates the syrinx accompanying wire encroachment in (B) sagittal and (C) axial views. (D) T2-weighted non-contrast imaging re-demonstrating the pseudomeningocele (arrow) in this patient.

At his initial presentation, the patient was found to have pseudoarthrosis at the initial cervical repair site, spasticity of the left lower extremity, and diffuse weakness of the left upper extremity to the point that he was only able to overcome gravity (i.e. graded 3/5). The patient was also found to exhibit pronator drift of the left upper extremity on examination. The patient’s right upper and lower extremities were neurologically intact. Reflexes were 2+ throughout, and his sensorium was normal on exam. A cervical computed tomography (CT) scan without contrast demonstrated sublaminar wiring between C1 and C2 with the encroachment of the upper cervical wire into the spinal canal with concern for dural and cord penetration as shown in Figure 1. A magnetic resonance imaging (MRI) scan of his cervical spine confirmed dural and cord penetration by the wire associated with an eccentric left-sided syrinx at C2 as shown in Figure 2. A pseudomeningocele can also be seen.

**Figure 3:**
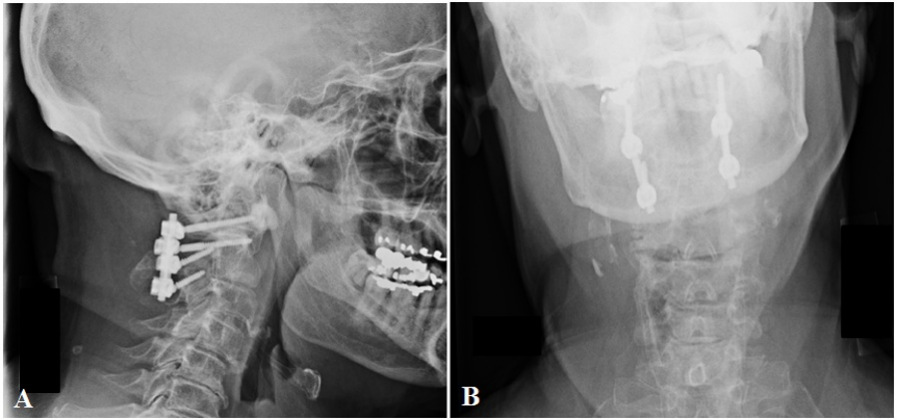
Postoperative X-ray Scans Postoperative X-rays (A, B) demonstrating removal of the sublaminar wires and proper hardware placement with fusion of cervical levels C1 and C2. Fusion was achieved using C1 lateral mass screws, C2 pars interarticularis screws, and rods.

The patient underwent operative removal of the broken sublaminar wires, repair of the durotomy, and posterior fusion with C1 lateral mass screws, C2 pars interarticularis screws, and rods. Image guidance was used for screw placement. Intraoperatively, spinal fluid was noted to be coming from areas not immediately adjacent to the dura. Dissection was continued until all potential sources of leakage were repaired and all wiring from the previous operation was accounted for and removed. Intraoperative and postoperative imaging showed proper hardware placement (Figure 3).

Following surgery, the patient was discharged to a rehabilitation center for continued intensive physical and occupational therapy. At his six-month follow-up visit, examination of the patient’s left upper extremity revealed nearly normal strength (4+/5). His previously described dysesthesias had improved, and he denied continued lower extremity spasms. He reported a return to baseline function at his yearly follow-up visit.

## Discussion

In this study, we present a case where sublaminar wiring for cervical stabilization resulted in myelopathy secondary to cord penetration by one of the failed wires. The reported risks of using sublaminar wiring vary considerably in the literature. In one study, there were no reported wire failures in a case series of 245 patients [7]. In a second study, five patients experienced neurological decline following cervical fixation with sublaminar wiring, corresponding to an overall complication rate of 7% [8]. The neurologic decline in this series of patients was associated with anterior bowing of the wires following fixation [8]. In reviewing the literature, we found two cases specifically involving transdural intramedullary migration of sublaminar wires [9-10]. In the first case report, intramedullary migration of a broken wire fragment occurred following occiput to C2 cervical fixation [9]. In the second case report, cervical transdural herniation occurred through an iatrogenic durotomy from a fractured sublaminar wire [10].

## Conclusions

The technique of sublaminar wire passage for spinal fixation is not without the risk of neurological decline. Our case illustrates the importance of imaging, especially in the setting of new, unexplained neurological deficits as previously stable constructs can fail. This case also demonstrates that reoperation for correction in these rare cases may be beneficial since patients may have the potential to improve following the removal of the compromised device.
